# Supplementary material for: Identification and analysis of Chrysanthemum nankingense NAC transcription factors and an expression analysis of OsNAC7 subfamily members
Source: PeerJ. 2021 May 26;9:e11505. doi: 10.7717/peerj.11505 (PMC8164415; doi:10.7717/peerj.11505)
Supplement: Supplemental Information 2 [file peerj-09-11505-s002.docx]

>CHR00069684-RA

ATGTCTGAAGAGGAAATGAGTTTATCCGTAAATGTTAATGGTCAATCGAAAGTGCCACCTGGTTTTCGGTTTCACCCTACGGAGGAAGAGCTTCTTCACTATTACTTGAGGAAGAAAGTTGCATATGAAAAGATCGATCTTGATGTTATTCGTGATGTTGATCTCAACAAGCTTGAACCCTGGGATATACAAGAGAAATGTAAAATAGGATCTACTCCACAAAATGATTGGTACTTCTTTAGTCATAAAGATAAGAAGTACCCTACGGGAACCAGAACGAATCGTGCGACTGCTGCTGGTTTCTGGAAAGCAACAGGCCGGGATAAGGTCATCTATAGTAGCGTTACAAGAATTGGTATGAGGAAAACACTTGTATTCTACAAAGGGAGAGCACCTCATGGCCAGAAATCTGATTGGATAATGCATGAATATCGGTTGGACGATAATACAATTGCGAATCAAGATGCATGTGGTCCAAACTTGTGTGATTCAGCTCAAGAAGATGGTTGGGTGGTGTGTCGTGTTTTCAAGAAGAAAAACTACCACAAATCTCTAGAAAGCCCCCAAAGATCGTTATCAGGCTCCATGGATTCCAGAACACAGTTACAATCGTTAAACAAAGACGGTGTTCTTGATCAATTACTCATGTACATGGGTAGCAATAGGTCATGCAAGCAAGAGATTGAGTCTTTAACTGCAAATCACAATGTTATGCAGCAGTTGGTGAGTCCTATTAACGAACGATTCTACCAACTCCCAAGGCTCGATAGTCCCACAATGACTATGTCCCCGCACTACAGCTCAGTTTCTGCAAACTTCAATCAAGAATTGAGTTTCAAGCCCCATGCAACTGACTTCCTGACAGAAGCTGATCAACCAACGAATATGGACCATGACTCTCGTGAACGTCTTGATAATTGGGCAGATCTTGAAAGGCTCGTGGCCACTCCACTTAACGTTCAAGTCGACTCTTCTAAGCAATTATACTCTTGTTATGGTGAACCAAATGAAAACTTCTGTTTCTCACTTGACAATGATGAGCAAGAACCACCACATCTTGGTGATTCAACCACAACCGGTAGAGCAAAGCACATAGTATACACGAGTGAGATAGACCTATGGAGCTTCGCTCAATCATCTTCATCTTCGTCATCCCCGGATCCATTATGTCACTTGTCAGTATAA
