# Supplementary figures and images for: Identification and analysis of Chrysanthemum nankingense NAC transcription factors and an expression analysis of OsNAC7 subfamily members
Source: PeerJ. 2021 May 26;9:e11505. doi: 10.7717/peerj.11505 (PMC8164415; doi:10.7717/peerj.11505)

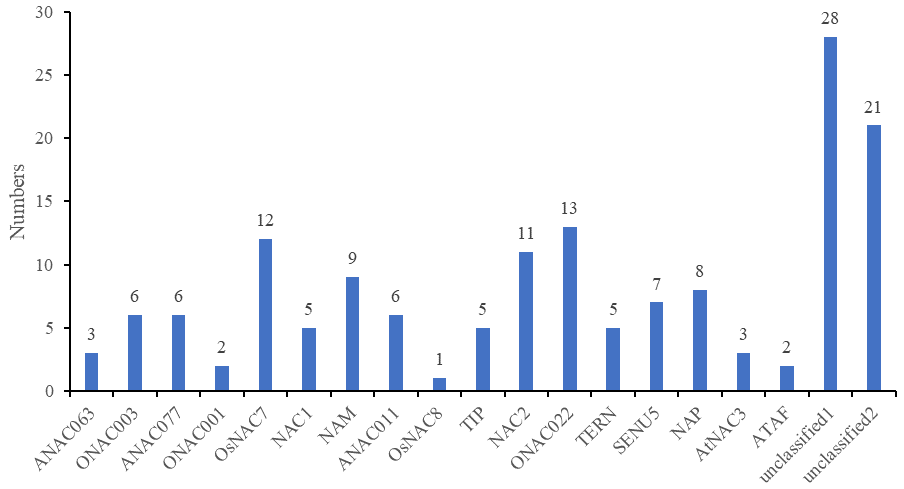

Supplement: Supplemental Information 4 — The x-coordinate shows the name of the subfamily and the y-coordinate shows the number of genes. [file peerj-09-11505-s004.png]

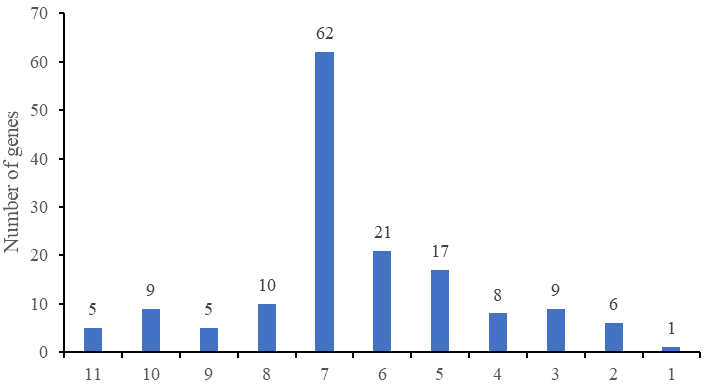

Supplement: Supplemental Information 5 — The x-coordinate represents the serial number of motifs, and the y-coordinate represents the number of genes. [file peerj-09-11505-s005.png]

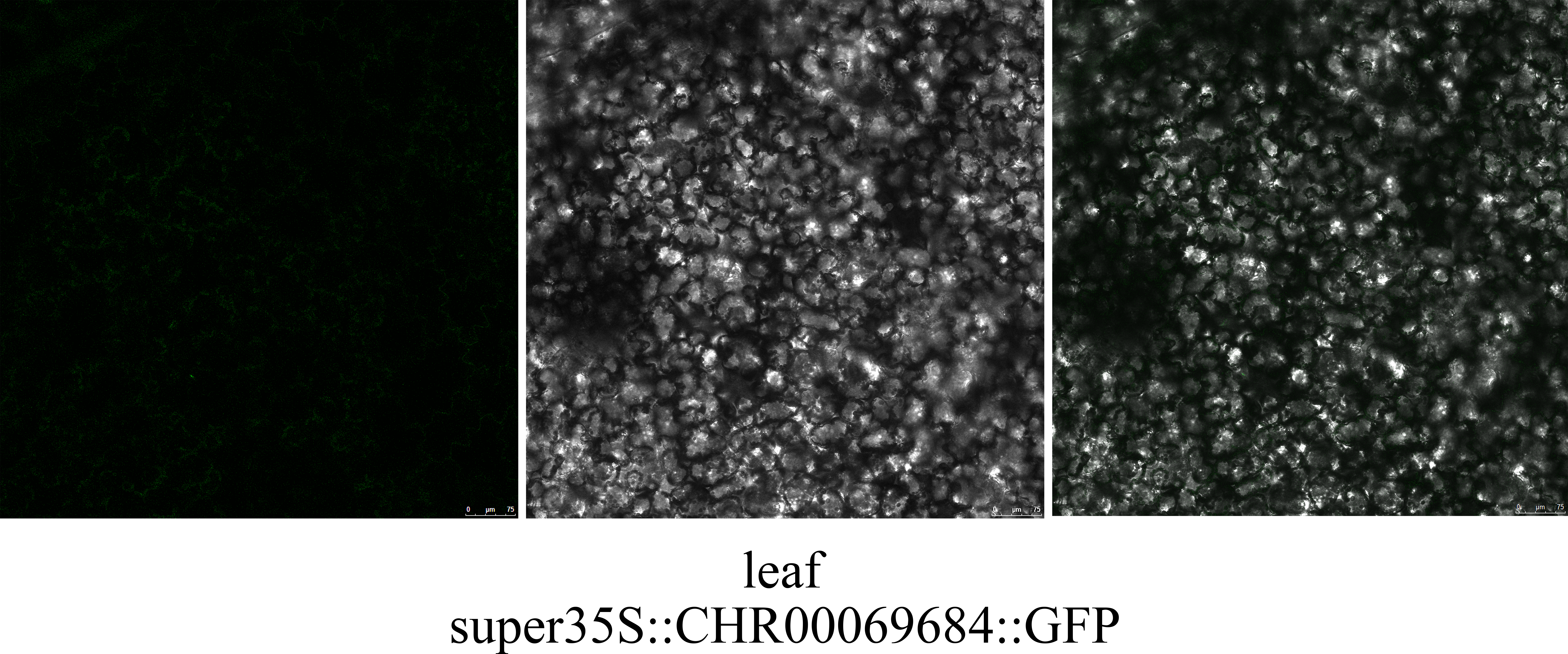

Supplement: Supplemental Information 6 [file peerj-09-11505-s006.png]
